# IFN-γ enhances protective efficacy against *Nocardia seriolae* infection in largemouth bass (*Micropterus salmoides*)

**DOI:** 10.3389/fimmu.2024.1361231

**Published:** 2024-03-13

**Authors:** Ruying Yu, Weixiang Zhang, Penghui Yu, Jiancheng Zhou, Jianguo Su, Gailing Yuan

**Affiliations:** ^1^ Department of Aquatic Animal Medicine, College of Fisheries, Huazhong Agricultural University, Wuhan, China; ^2^ College of Fisheries, Zhejiang Ocean University, Zhoushan, China; ^3^ Jiangsu DABEINONG Group (DBN) Aquaculture Technology Co. LTD, Huai’an, China

**Keywords:** *Micropterus salmoides*, interferon-γ, monocytes/macrophages, *Nocardia seriolae*, granuloma

## Abstract

**Introduction:**

*Nocardia seriolae* adversely impacts a diverse range of fish species, exhibiting significant pathogenic characteristics that substantially impede the progress of aquaculture. *N. seriolae* infects in fish has a long incubation period, and clinical symptoms are not obvious in the early stages. There is presently no viable and eco-friendly approach to combat the spread of the disease. According to reports, *N. seriolae* primarily targets macrophages in tissues after infecting fish and can proliferate massively, leading to the death of fish. Interferon-gamma (IFN-γ) is a crucial molecule that regulates macrophage activation, but little is known about its role in the *N. seriolae* prevention.

**Methods:**

IFN-γ was first defined as largemouth bass (*Micropterus salmoides*, MsIFN-γ), which has a highly conserved IFN-γ characteristic sequence through homology analysis. The recombinant proteins (rMsIFN-γ) were obtained in *Escherichia coli* (*E. coli*) strain BL21 (DE3). The inflammatory response-inducing ability of rMsIFN-γ was assessed *in vitro* using monocytes/macrophages. Meanwhile, the protective effect of MsIFN-γ *in vivo* was evaluated by *N. seriolae* infection largemouth bass model.

**Results:**

In the inflammatory response of the monocytes/macrophages activated by rMsIFN-γ, various cytokines were significantly increased. Interestingly, interleukin 1β (IL-1β) and tumor necrosis factor alpha (TNF-a) increased by 183- and 12-fold, respectively, after rMsIFN-γ stimulation. rMsIFN-γ improved survival by 42.1% compared with the control. The bacterial load in the liver, spleen and head kidney significantly decreased. rMsIFN-γ was also shown to better induce increased expression of IL-1β, TNF-α, hepcidin-1(Hep-1), major histocompatibility complex I (MHCI), and MHC II in head kidney, spleen and liver. The histopathological examination demonstrated the transformation of granuloma status from an early necrotic foci to fibrosis in the infection period. Unexpectedly, the development of granulomas was successfully slowed in the rMsIFN-γ group.

**Discussion:**

This work paves the way for further research into IFN-γ of largemouth bass and identifies a potential therapeutic target for the prevention of *N. seriolae*.

## Highlights

- Recombinant MsIFN-γ improves the survival rate of largemouth bass infected with *N. seriolae*.
- MsIFN-γ delays the progression of granulomas.
- MsIFN-γ enhances the ability of largemouth bass to resist *N. seriolae* infection by activating macrophages.

## 1 Introduction

Largemouth bass (*Micropterus salmoides*) is globally recognized as one of the most economically significant fish species in aquaculture (1). It was introduced into China in the 1980s and has expanded to become a significant economic fish in China’s freshwater aquaculture industry (2). However, the rate of severe diseases increased with the scale of largemouth bass farming, causing significant economic losses. Bacterial disease has emerged as one of the important limiting factors due to its high fatality rate and susceptibility to drug resistance. Traditional antibiotics have been historically used for treating bacterial infections. However, the use of antibiotics alone cannot resolve all complications, and the emergence of drug-resistant strains and antibiotic residues deserves further attention. *Nocardia seriolae* has become the main pathogen causing sarcoidosis in largemouth bass. In recent years, it has posed a serious threat to the economic growth of largemouth bass production (3). In addition, other fish have also been affected by *N. seriolae* in the development of aquaculture, including snakehead (*Channa argus*), derbio (*Tranchinotus ovatus*) (4), Atlantic salmon (*Salmo salar*) (5), and freshwater eel (*Anguilla japonica*) (6), among others.

*N. seriolae* is a Gram-positive branched filamentous bacteria belonging to intracellular parasites/intracellular amastigotes (7, 8). The typical feature of bacterial infection by *N. seriolae* is systemic and chronic granuloma (7). After infection with *N. seriolae*, fish show obvious ulceration on the body surface, and the tissue (e.g., head kidney, spleen, liver, or muscle) forms white nodules of varying numbers (9). Furthermore, *N. seriolae* has a long incubation period, a slow infection process, and a high infection rate and mortality. When fish are challenged with *N. seriolae*, the tissue-resident macrophages are activated to release enormous cytokines (e.g., IL-1β and TNF-α, among others) and can exert phagocytosis (10, 11). In the infection stage, granulomata, as an immune protective mechanism, are formed by the interaction between *N. seriolae* and cells (12). Granulomas recruit inflammatory cells such as monocytes/macrophages, lymphocytes, and granulocytes to aggregate. After the macrophages phagocytose bacteria, the complex composition of the bacterial cell walls hinders complete bacterial decomposition, even with a large number of inflammatory cells present. Consequently, *N. seriolae* eventually become surrounded by connective tissue and epithelial cells, resulting in the partial control of the bacteria. This incomplete inhibition allows for further tissue damage to occur. Antibiotics are frequently employed to limit the spread of *N. seriolae*; however, due to the complicated structures of granulomas, antibiotics cannot successfully penetrate to eradicate the pathogens. In addition, although vaccines may offer better prevention and disease control options, there is currently no licensed vaccine available against *N. seriolae*, and ongoing vaccine development remains incomplete (8). There are no validated response measures (13). Thus, findings of these phenomena could prompt the search for more valid methods to address these issues.

Interferon gamma (IFN-γ) belongs to the type II interferons (IFNs) and is a multifunctional cytokine widely distributed in various vertebrates. The conserved IFN-γ sequence (nuclear localization sequence) has been identified in many species, including humans, mice, and fish. Unlike the IFN-γ in other mammals, some fish species also possess a second IFN-γ subtype, termed IFN-γrel, which is different from the classical IFN-γ (14). IFN-γ has been identified and proven to play a major role in both innate and adaptive immune responses. It can be secreted by numerous cells, including natural killer (NK) cells, T helper 1 (Th1) cells, NK T cells, monocytes, and macrophages, among others (15). Thus, IFN-γ can act more quickly and directly on immune cells. It not only exhibits antiviral effects, stimulating the expression of major histocompatibility complex I (MHC I) and MHC II in response to antigen presentation, but also encourages Th1 immune responses, activates inflammatory responses, and contributes to antimicrobial effects (16). As the primary target cells of IFN-γ are macrophages, IFN-γ has become an extremely crucial regulator of macrophage activation. The recombinant IFN-γ and IFN-γrel of gold fish (*Carassius auratus*) (17) and the IFN-γ of the large yellow croaker (*Larimichthys crocea*) have been shown in studies to greatly increase macrophage phagocytic activity (17, 18). Interleukin 1β (IL-1β), tumor necrosis factor alpha (TNF-α), interleukin 6 (IL-6), and other pro-inflammatory cytokines are secreted by activated macrophages that can hinder pathogenic invasion. Previous research in our laboratory demonstrated that the IFN-γ2 of grass carp (*Ctenopharyngodon idella*) can effectively promote the expression levels of IL-1β, TNF-α, IL-6, and inducible nitric oxide synthase (iNOS), which are typical indicators of classically activated macrophages, in monocytes/macrophages (19). *N. seriolae* is quickly absorbed into tissues through the blood circulation and is widely distributed throughout the whole body. Injection with green fluorescent protein-labeled *N. seriolae* into transparent tiger barb (*Puntius tetrazona*) showed macrophages engulfing large amounts of bacteria (20). Bacteria proliferate in macrophages for a long time before aggregation and death, forming granulomas. Thus, it was presumed that IFN-γ has a positive protective effect in the treatment of *N. seriolae* infection through the activation of monocytes/macrophages.

Based on the above information, this study describes the protein structure of *Micropterus salmoides* IFN-γ (MsIFN-γ) and examines the homology of the IFN-γ gene in largemouth bass. Recombinant MsIFN-γ (rMsIFN-γ) protein was prepared using an *Escherichia coli* expression–purification system. The expression levels of pro-inflammatory cytokines in monocytes/macrophages were measured to assess the function of MsIFN-γ *in vitro*. In a largemouth bass infected with *N. seriolae* model, exogenous supplementation of rMsIFN-γ by intraperitoneal injection increased the survival rate, reduced the tissue bacterial load, and decreased tissue damage. In conclusion, our work demonstrates the essential role of IFN-γ in the treatment of *N. seriolae*, offers guidance for a healthier and more productive method, and encourages sustainable development of aquaculture.

## 2 Materials and methods

### 2.1 Fish and bacteria

Largemouth bass (30.0 ± 3.0 g) were purchased from the Wuhan Taihongyuan Agricultural Technology Co., Ltd. Hubei Province, China. The fish were randomly distributed into nine tanks with approximately 50 fish per tank. The fish were adapted to a circulating freshwater system at 25°C and fed normally daily. The Animal Experiment Ethics Committee of Huazhong Agricultural University in China approved all animal experiments, which adhered to the National Guidelines for the Care and Use of Laboratory Animals (HZAUFI-2022-0015).

The utilized *N. seriolae* was generously provided by Dr. Junfa Yuan of the College of Fisheries, Huazhong Agricultural University. Cultivation of *N. seriolae* was carried out in brain–heart infusion (BHI) broth at 28°C with shaking. The *E. coli* strains DH5α and BL21(DE3), provided by Shanghai Weidi Biotechnology Co., Ltd., were used for plasmid construction and recombinant protein expression. The absorbance at 600 nm (OD_600_ = 0.8–1.0) of the growing bacteria was measured using a spectrophotometer (UV-1700, Pharma Spec; Shimadzu, Columbia, MD, USA).

### 2.2 Characterization of MsIFN-γ

We found the amino acid sequence of IFN-γ (GenBank: XP_038563402; https://www.ncbi.nlm.nih.gov/protein/1951417769) in largemouth bass at the National Center for Biotechnology Information (NCBI; https://www.ncbi.nlm.nih.gov/home/). The signal peptide of MsIFN-γ was predicted by SignalP-5.0 (https://services.healthtech.dtu.dk). The three-dimensional (3D) model predictions of MsIFN-γ were performed with the Iterative Threading Assembly Refinement (https://zhanglab.ccmb.med.umich.edu/I-TASSER/). In order to predict the protein model and net charge distribution model, the protein structure was entered into PyMOL (PyMOL Molecular Graphics System; Schrödinger, New York, NY, USA). The amino acid sequences of IFN-γ from six Perciformes fish were compared using the muscle in MEGA 7. Similarly, a neighbor-joining (NJ) tree of the type II IFNs of 27 vertebrates was established using the adjacency method, and 1,000 bootstraps were carried out.

### 2.3 Expression analysis of MsIFN-γ *in vivo*

Using specific primers, quantitative real-time PCR (qRT-PCR) was used to determine the tissue distribution of MsIFN-γ. Normalization of each experiment was performed using the *β-actin* gene. Various tissue samples were collected from healthy largemouth bass (*n* = 8). Following the manufacturer’s instructions, the total RNA of largemouth bass tissue samples was extracted using TRIzol reagent (RN0401; AiDLab, Beijing, China). Ultraviolet spectrophotometry and agarose gel electrophoresis (1.0%) were used to assess the RNA concentration and purity of the extracted RNA samples. Reverse transcription of total RNA and complementary DNA (cDNA) synthesis were carried out using the Vazyme cDNA Synthesis Kit (R223-01; Vazyme Biotechnology Co., Ltd., Nanjing, China) with a reaction volume of 20 μL. qRT-PCR was performed on the QuantStudio™ 6 Flex Real-Time PCR system (Thermo Fisher Scientific, Waltham, MA, USA). The PCR volume was 20 μL and contained 4 μL of the cDNA sample with 0.5 μL (10 μM) of each gene-specific primer, 10 μL of 2× Universal SYBR Green Fast qPCR Mix (RK21203; ABclonal Biotech Co. Ltd., Wuhan, China), and 5 μL of nuclease-free water. The PCR procedures consisted of 95°C for 3 min, 95°C for 15 s, 60°C for 15 s, and 72°C for 20 s, followed by 40 cycles in step 2. The housekeeping gene *β-actin* was used as an internal standard. The change in gene expression was determined using the 2^−ΔΔCT^ method after further comparison with the control group. **Supplementary Table S1** contains a list of the qRT-PCR primers that were created using Primer Premier 5.

### 2.4 Expression and purification of recombinant fusion MsIFN-γ

The total RNA of the largemouth bass liver was extracted using TRIzol reagent. The cDNA was then synthesized by qPCR (+gDNA wiper) of the total RNA with HiScript®II Q RT SuperMix in a reaction volume of 20 μL. The detailed procedure was the same as that described in *Section 2.3*. Based on the MsIFN-γ sequence (GenBank: XM 038707474.1) obtained from NCBI, Primer Premier 5.0 software was employed to generate the cDNA primers for areas with non-signal peptides. The open reading frame (ORF) of MsIFN-γ was confirmed by PCR. The PCR reaction volume was 20.0 μL, which consisted of 10.0 μL PowerPol 2× PCR Mix with Dye (RK20719; ABclonal, Wuhan, China), 8.0 μL ddH_2_O, 1.0 μL cDNA, 0.5 μL forward primer (MsIFN-γ-F), and 0.5 μL reverse primer (MsIFN-γ-R). The primers were synthesized by Tsingke Biotechnology Co., Ltd. (Wuhan, China). On the ProFlex™PCR system, MsIFN-γ was amplified using the following conditions: 95°C for 5 min, then 30 cycles of 95°C for 30 s, 58°C for 35 s, 72°C for 30 s, and finally prolonged for 10 min at 72°C. All PCR products were extracted from the gel using the DNA Gel Extraction Kit after detection on 1.0% agarose gel (Axygen, Union City, CA, USA). Subsequently, the purified PCR product was successfully constructed into the pET-32a vector with the *Bam*HI and *Xho*I restriction sites, which was further transformed into the *E. coli* DH5α. Positive clones were manually screened and sequenced by Tsingke Biotechnology Co., Ltd. (Wuhan, China).

The pET-32a–MsIFN-γ verified recombinant expression plasmid was converted into the *E. coli* DE3 and then incubated in Luria–Bertani (LB) medium at 37°C for 4 h. The protein expression was induced by the addition of IPTG (final, 1.0 mM) for 4 h at 37°C. In addition, as a negative control, the thioredoxin (Trx)-tagged protein carrying pET-32a was expressed in the same manner. Bacteria were then collected and centrifuged at 5,000 rpm for 15 min at 4°C. After reviving the bacteria with phosphate-buffered saline (PBS; pH=7.4), they were disrupted for 10 min using a high-pressure cell disruptor (880 MPa). The supernatant and pellet were collected following 1 h of centrifugation at 4°C and 12,000 rpm.

The protein aggregate pellet in the form of inclusion bodies was washed twice with buffers A, B, and C (50 mM Tris-HCl, 5 mM EDTA, and 1% Triton X-100, pH=8.0) and centrifuged at 12,000 rpm for 30 min to purify the recombinant MsIFN-γ proteins. Buffer D (0.1 M Tris-HCl, 10 Mm EDTA, and 8 M urea, pH=8.0) was further used, which contained high amounts of urea, in a thermostatic shaker at 37°C, 180 rpm for 1 h, followed by a centrifugation step to preserve the supernatant at 12,000 rpm for 30 min. After refolding the denatured protein, the urea content was gradually lowered in the external dialysate. Moreover, the Trx-tagged protein was expressed in the supernatant after high-speed centrifugation.

Proteins were purified by affinity chromatography using Ni-NTA resin (HZ1003-8; Huiyan Bio, Wuhan, China) according to the manufacturer’s protocol. After pre-equilibration with buffer A (50 mM Tris, 500 mM NaCl, and 1% glycerol, pH=8), the supernatant with the His tag was incubated overnight at 4°C with the Ni-NTA resin. The protein was washed with buffer B (50 mM Tris, 500 mM NaCl, 20 mM imidazole, and 1% glycerol, pH=8) and eluted with buffer C (50 mM Tris, 500 mM NaCl, 300 mM imidazole, and 1% glycerol, pH=8) from the resin. SDS-PAGE (12% gel; Biosharp, Hefei, China) was utilized to detect pure recombinant protein, while BCA kits (Solarbio, Beijing, China) were used to measure its content. Western blot and SDS-PAGE were used to verify the results. The level of endotoxin was measured using the Limulus Amebocyte Lysate (LAL) Kit (Beyotime, Shanghai, China), and Western blot and SDS-PAGE were used to verify the results. The protein was transferred into a 0.22-μm nitrocellulose (NC) membrane (BS-NC-22; Biosharp, Hefei, China) via a wet electroblotting technique. The membrane was blocked for 2 h at room temperature (RT) with TBST buffer containing 5% (*w*/*v*) skim milk before incubation with a mouse His-tagged monoclonal antibody (mAb) (diluted 1:3,000, AE003; ABclonal, Wuhan, China) for 2 h at RT with shaking. After three TBST washes, the membrane was incubated for 45 min at RT with a horseradish peroxidase (HRP)-labeled goat anti-mouse IgG (diluted 1:5,000, AS003; ABclonal, Wuhan, China). The membrane was once again washed with TBST before the signal was applied using a clear Western ECL substrate. Thereafter, the Amersham Imager 680 was employed to image the membrane.

### 2.5 Isolation, culture, and stimulation of largemouth bass head kidney monocytes/macrophages

As previously mentioned, the Percoll density gradient was used to separate the head kidney monocytes/macrophages of largemouth bass (P1644; Sigma, Shanghai, China) (21). The fish were anesthetized in MS-222 (886-86-2; Macklin, Shanghai, China) before the head kidney tissue was separated aseptically. The tissues were screened through a 100-μm screen in Leibovitz medium (L-15, L620KJ; BasalMedia, Shanghai, China) and mixed with 2.0% fetal bovine serum (FBS) and 200 IU/mL penicillin plus streptomycin. The resultant cell suspension was centrifuged at 400 × *g* for 30 min using Percoll with a 34%/51% density gradient. The monocytes/macrophages were then collected at the layered liquid level, washed twice with L-15 at 400 g for 10 min at 4°C, then resuspended at 1 × 10^7^ cells/mL in L-15 medium with 10% FBS and grown at 28°C.

In order to stabilize the cells, the isolated head kidney monocytes/macrophages were planted at a density of 1 × 10^7^ in six-well plates. These cells were restimulated *in vitro* with rMsIFN-γ (500 ng/mL) and Trx (500 ng/mL) for 6 h at 28°C. An equivalent volume of PBS served as a blank control. Using trypsin, adherent cells were separated after 6 h of incubation. The cell pellets were then collected, rinsed three times with PBS, and total RNA was extracted using the TRIzol method. qRT-PCR was performed to determine the expression levels of IFN-γ, IL-1β, TNF-α, MHC I, MHC II, hepcidin-1 (Hep-1), and IL-6. Each experiment was repeated three times.

### 2.6 Fish infection, tissue bacterial load, and cytokine expression levels

Before the start of the formal experiments, lethal concentrations (LC_50_ values) of *N. seriolae* were determined by dilution into four series (i.e., 5.0 × 10^6^, 1.0 × 10^7^, 2.0 × 10^7^, and 4.0 × 10^7^ CFU/mL). Each group consisted of 25 fish. Fish groups were injected intraperitoneally with bacteria, and mortality was recorded for 14 days. The LC_50_ concentrations for *N. seriolae* were determined by Probit regression analysis using the IBM SPSS Statistics package (22). The LC_50_ value for *N. seriolae* in largemouth bass was 1.0 × 10^7^ CFU/mL. Fish were randomly divided into three experimental groups, with three replicates in each group (50 × 3). The animals were intraperitoneally injected at 6.0 μg/fish with rMsIFN-γ, Trx, or PBS (negative control). After 6 h, all experimental fish were intraperitoneally injected with *N. seriolae* at a concentration of 1.0 × 10^7^ CFU/mL (OD_600_ = 0.24). All of the infected fish were monitored every 8 h for clinical signs and mortality. On the third day of bacterial injection, the same dose of protein was again injected intraperitoneally as booster immunization.

The tissues (liver, spleen, and head kidney) were collected on day 0 (D0), D3, D7, and D14 post-bacterial infection (*n* = 9 in each group). The tissues were immediately submerged in TRIzol reagent. According to the previous description (see *Section 2.3*), the total messenger RNA (mRNA) was extracted from these tissues and converted to cDNA for qRT-PCR. The tissue bacterial load was determined by quantifying the copies of the *N. seriolae* 16S rRNA using qRT-PCR, as previously described (23). The 16S rRNA specific primers of *N. seriolae* (forward: TGCTACAATGGCCGGTACAGAG; reverse: TTCACGAGGTCGAGTTGCAGAC) were used for amplification. To assess the host immune response, changes in the expression levels of the cytokines (i.e., IFN-γ, IL-1β, TNF-α, MHC I, and MHC II) in these tissues were analyzed using qRT-PCR.

### 2.7 Clinical symptoms and histopathological examination

To better observe the infection period of *N. seriolae* on largemouth bass, the clinical symptoms were photographed using a digital camera. On D7 and D14, largemouth bass from different treatment groups were randomly selected and quickly anesthetized using MS-222. These fish were dissected after anesthesia and the head kidney, liver, and spleen were removed. The clinical symptoms were photographed to observe differences.

The surface of randomly caught largemouth bass was examined prior to separation of the tissues, and the visceral tissue lesions following *N. seriolae* infection were dissected and examined. To make the choice of appropriate tissue sections easier in the future, infected fish was compared to a healthy largemouth bass in order to identify the primary lesions and areas of disease. Dissection of the liver, spleen, and head kidney was followed by a 24-h fixation in 4% paraformaldehyde (PFA), dehydration, paraffin embedding, and sectioning, constituting the specific pathological sectioning procedure. The sectioned sample (4 μm) was placed on a slide treated with aminopropyl triethoxysilane. The sections were rehydrated, stained with hematoxylin and eosin (HE), neutral glue was applied, and photographs were taken after deaffinization in xylene.

### 2.8 Statistical analysis

Data were reported as average ± SEM prepared and statistically analyzed using GraphPad Prism 8.0 software. The Kruskal–Wallis test was used for each experimental group relative to the control group, followed by Dunn’s multiple comparison (adjusted by Bonferroni) to determine significance. Significance was expressed as *p < 0.05, **p < 0.01, and ***p < 0.001.

## 3 Results

### 3.1 Analysis of the amino acid structure and phylogenetic relationship

**Figure 1:**
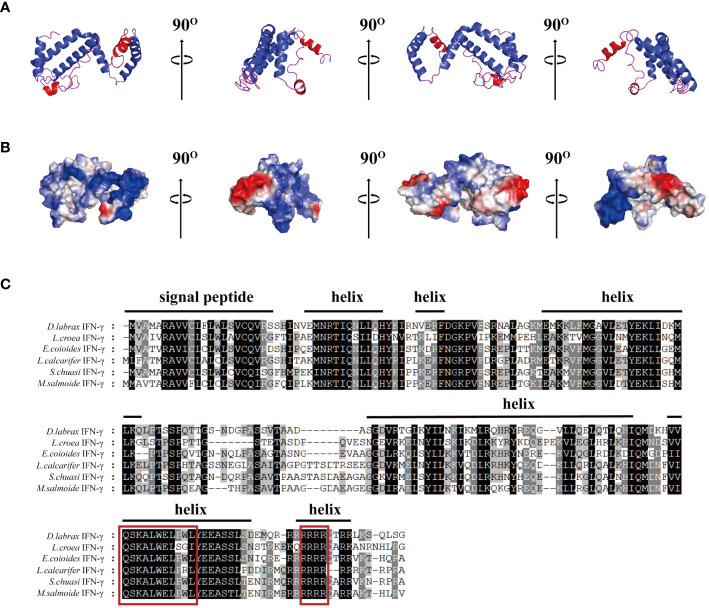
Bioinformatics analysis of the interferon gamma of *Micropterus salmoides* (MsIFN-γ). **(A)** Homologous modeling to obtain colored protein bands of MsIFN-γ. **(B)** Three-dimensional model of MsIFN-γ. The positively charged region is ***blue***, the negatively charged region is ***red***, and the hydrophobic charge region is ***white***. **(C)** Multiple sequence alignment of IFN-γ proteins in Perciformes. Identical amino acids are represented by a ***black shade***, while similar amino acids are represented by a ***shade of gray***. The putative signal peptide and helices of MsIFN-γ are highlighted by ***solid lines*** above the alignment. In the ***red frame*** are the IFN-γ feature sequences and the nuclear localization signal (NLS) sequences.

The 3D modeling results showed that the IFN-γ of largemouth bass consisted of six alpha helices and partial irregular curls (**Figure 1A**). Most of the cationic amino acids (blue) were adjacent, aggregate, formed cationic clusters, and were exposed to the surface. Two or three clusters of hydrophobic amino acids (red) were scattered around them (**Figure 1B**). The signal peptide of MsIFN-γ was predicted using SignalP-5.0, in addition to the 23 amino acids of the signal peptide. Thus, MsIFN-γ consisted of a 23-amino acid signal peptide and a 182-amino acid mature peptide with a molecular weight of about 20.9 kDa.

**Figure 2:**
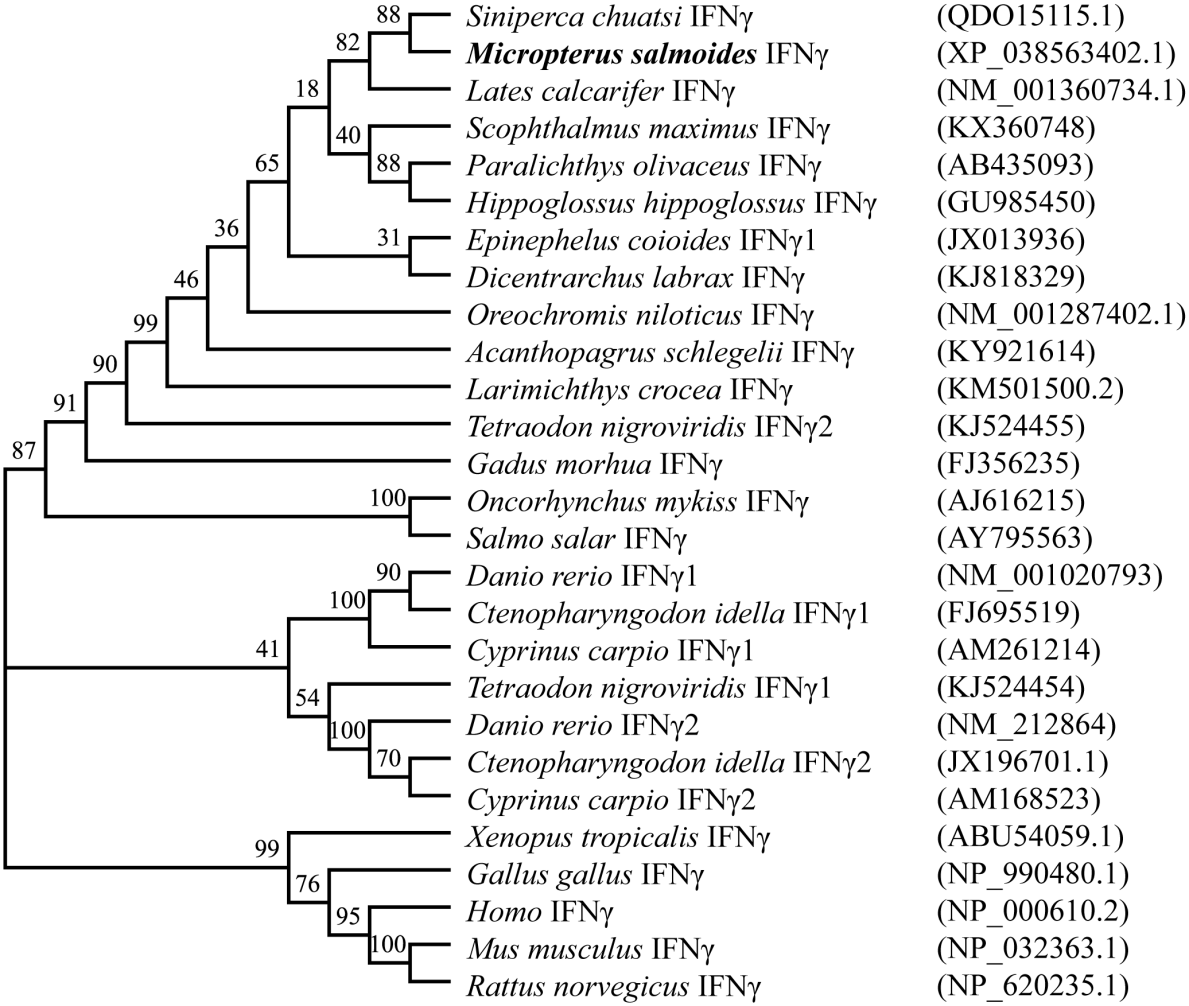
Interferon gamma (IFN-γ) phylogenetic tree of vertebrates. Bootstrap (1,000) neighbor-joining (NJ) tree was constructed using a minimum composite likelihood model by protein sequence alignment of 27 vertebrate IFN-γ species in MEGA 7 software.

The results of various sequence alignments showed that the type II interferon of the six Perciformes fish had the terminal region of the C-terminal containing comparatively conserved nuclear localization signal (NLS) sequences (continuous lysine or arginine) and signature sequence ([I/V]-Q-X-[K/Q]-A-X2-E-[L/F]-X2-[I/V]) (**Figure 1C**). Despite the more conserved amino acid regions, MsIFN-γ is not very similar to the other remaining fishes. The highest homology was to the mandarin fish (*Siniperca chuatsi*) IFN-γ, with 76.97%. The lowest homology was to the European sea bass (*Dicentrarchus labrax*), with only 53.33%. A study of the type II IFNs of 27 vertebrates discovered that MsIFN-γ and mandarin fish belong to the same subclade. It was relatively clustered with the type II IFNs of the orders Perciformes and Pleuronectiformes. However, it forms distinct clades from Cypriniformes, avians, and mammals. It is worth mentioning that MsIFN-γ is most closely related to the type II IFNs of marine teleost fishes and belongs to IFN-γ (IFN-γ2) (**Figure 2**).

### 3.2 Expression, identification, and biological activity of rMsIFN-γ

**Figure 3:**
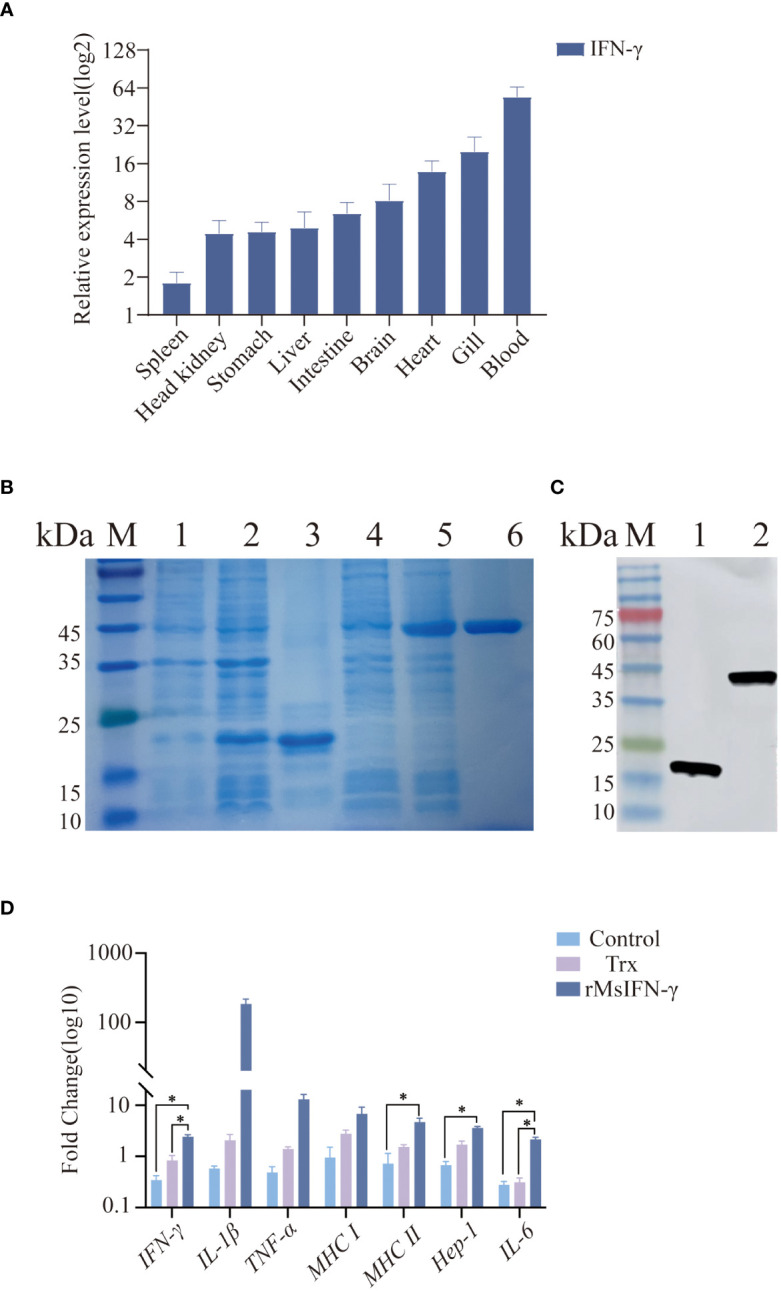
Tissue distribution and viability assay of the interferon gamma of *Micropterus salmoides* (MsIFN-γ). **(A)** MsIFN-γ expression analysis of organizations. Total RNA was extracted from normal largemouth bass spleen, head kidney, stomach, liver, intestine, brain, heart, gills, and blood and reverse-transcribed into cDNA as a template for real-time PCR detection. ***β-actin*** was used as an internal reference. Each experiment was performed in eight replicates. **(B)** SDS-PAGE to detect protein expression. ***M***, Standard protein molecular weight marker; ***1***, Uninduced pET-32a protein; ***2***, Induced pET-32a protein; ***3***, Purified thioredoxin (Trx) protein; ***4***, Uninduced pET-32a–MsIFN-γ protein; ***5***, Induced pET-32a–MsIFN-γ protein; ***6***, Purified MsIFN-γ protein. **(C)** His antibody immunoblotting assay. ***M***, Standard protein molecular weight marker; ***1***, Purified Trx protein; ***2***, Purified recombinant MsIFN-γ (rMsIFN-γ) protein. **(D)** qRT-PCR method to detect the expression of immune genes by rMsIFN-γ on monocytes/macrophages. All results were normalized to the ***β-actin*** levels. Results are expressed as the average ± SEM of three replicates. *p<0.05.

In order to explore the immune function of MsIFN-γ *in vivo*, we investigated the expression of IFN-γ in various tissues of largemouth bass. The results showed MsIFN-γ having the highest expression levels in the blood. This was followed by the gill and the heart, with the lowest expression in the spleen (**Figure 3A**).

After amplification by specific PCR primers, agarose gel electrophoresis showed that the band size 558 bp was consistent with the target band size. MsIFN-γ with 6× His tag was effectively expressed by *E. coli* DE3 stimulated with 1.0 mM IPTG after IFN-γ gene cloning to the expression vector pET-32a. The Trx-tagged protein (pET-32a empty vector) was produced at the same time as a control. MsIFN-γ was verified by SDS-PAGE and Western blotting. The Trx-tagged protein had an estimated size of 19.8 kDa, which was consistent with the band of interest. Despite the target protein being predicted to be around 40.8 kDa, rMsIFN-γ with an interest band at around 45.0 kDa was more obvious than before induction (**Figure 3B**). The effective expression of rMsIFN-γ was shown by the His-tagged antibodies’ detection of unique bands. After purification, a single band with clear and high purity was obtained, which can be used for subsequent experiments (**Figure 3C**). The purified Trx protein (0.26 EU/mL) and the rMsIFN-γ protein (0.20 EU/mL) had very low lipopolysaccharide (LPS) levels.

We examined whether rMsIFN-γ is bioactive, i.e., it activates monocytes/macrophages and downstream immune genes. The cells were treated with 500 ng/mL of the Trx-tagged protein and rMsIFN-γ (**Figure 3D**). Compared with the Trx group and the control group, the expression levels of IFN-γ, MHC II, Hep-1, and IL-6 after 6 h stimulation were significantly upregulated in the rMsIFN-γ group (*p* < 0.05). The expression levels of IL-1β and TNF-α in the rMsIFN-γ group were 183- and 12-fold higher than those in the control group, respectively. In summary, MsIFN-γ can effectively activate monocytes/macrophages and regulate the expression of pro-inflammatory cytokines, which is conducive to monocytes/macrophages exerting immunomodulatory effects.

### 3.3 rMsIFN-γ effectively alleviate the infection of *N. seriolae*

**Figure 4:**
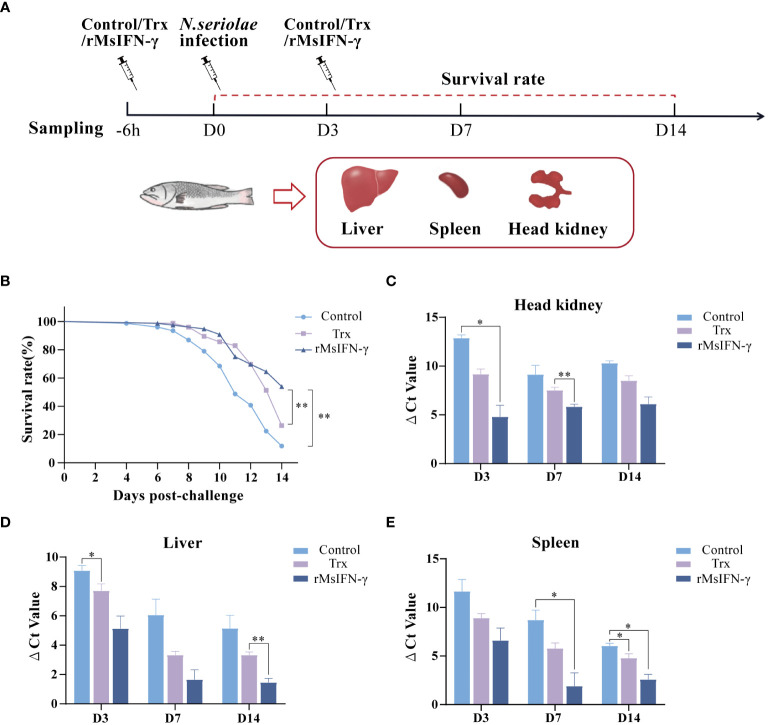
Survival rate and tissue bacterial load of *Nocardia seriolae* infection in different treatment groups. **(A)** Schematic diagram of protein injection, bacterial infection, and sampling time. **(B)** Survival monitoring during 14 days of infection (*n* = 150). **(C–E)** The head kidney **(C)**, liver **(D)**, and spleen **(E)** were collected on day 3 (D3), D7, and D14 after infection. qRT-PCR was used to determine the tissue bacterial load (*n* = 3). *p < 0.05; **p < 0.01.

We also determined whether rMsIFN-γ exerts an immunomodulatory effect to prevent bacterial infection. The initial intraperitoneal injection of rMsIFN-γ was found to boost the immune response 6 h before injection of *N. seriolae*. The second immunization injection of rMsIFN-γ on D3 of *N. seriolae* infection was used to boost immunity. PBS and Trx-tagged protein injections served as controls (**Figure 4A**). Within 14 days of infection with *N. seriolae*, the first 4 days did not record any deaths, whereas an extensive number of deaths occurred in largemouth bass during the late stage of infection. The survival rate (53.94%) in the rMsIFN-γ group was extremely significant (*p* < 0.01), which was higher than that in the control (11.84%) and the Trx group (26.31%) (**Figure 4B**). The liver, spleen, and head kidneys were the three organs that had an obvious onset after *N. seriolae* infection. Examination of the bacterial load in these organs also showed that the rMsIFN-γ group had a lower load compared with the control and the Trx group (**Figures 4C–E**). Bacterial infection of the head kidney and spleen occurred earlier relative to that of the liver. The bacterial burden in the head kidney remained at relatively high levels (**Figure 4C**). Thus, rMsIFN-γ had greater impact on lowering bacterial burden in the later phases of liver and spleen infection.

### 3.4 rMsIFN-γ effectively reduces the clinical symptoms of *N. seriolae* infection in largemouth bass

**Figure 5:**
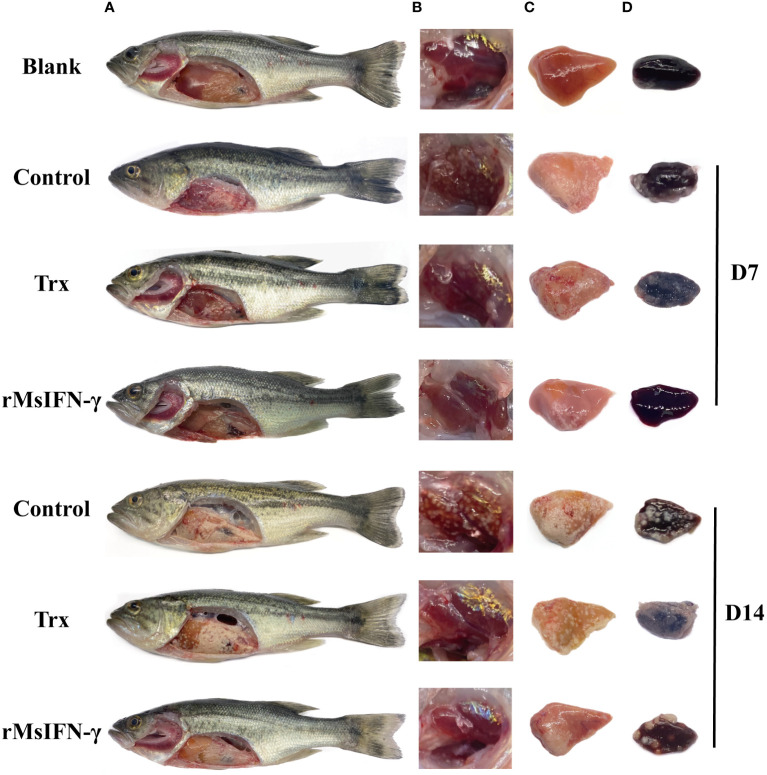
Anatomical observation of body surface changes and clinical symptoms of diseased largemouth bass. Fish from different groups were treated on day 7 (D7) and day 14. There was no ulceration on the body surface, and white nodules were found on the surface of the liver and pyloric caecum in the abdominal cavity **(A)**; head kidneys **(B)**, liver **(C)** and spleen **(D)** are distributed with white nodules.

After injection with *N. seriolae*, largemouth bass developed anorexia and slow swimming in the early stage. The infected fish occasionally had white nodules the size of a pinpoint and pale in the gill filaments. Following autopsy, it was observed that 1–2 mm white nodules were visible in the liver, spleen, head kidney, and pyloric caecum, among other sites. Moreover, the number of head kidney nodules was higher. On D14 of infection, the size of the white nodules on the tissues greatly increased, basically covering the entire tissues. However, none of the three groups of fish displayed evident surface lesions within 14 days of infection (**Figure 5A**). The number of head kidney nodules in the rMsIFN-γ group was low compared to that in the control and Trx groups. On visual inspection, the largemouth bass surviving on D14 were basically free of nodules (**Figure 5B**). However, the onset of symptoms in fish in the rMsIFN-γ group was also milder than that in the control group with regard to the liver and spleen (**Figures 5C, D**). At this point, it could be hypothesized that rMsIFN-γ successfully delayed the invasion of *N. seriolae*.

### 3.5 rMsIFN-γ upregulates the expression levels of immune-related genes

**Figure 6:**
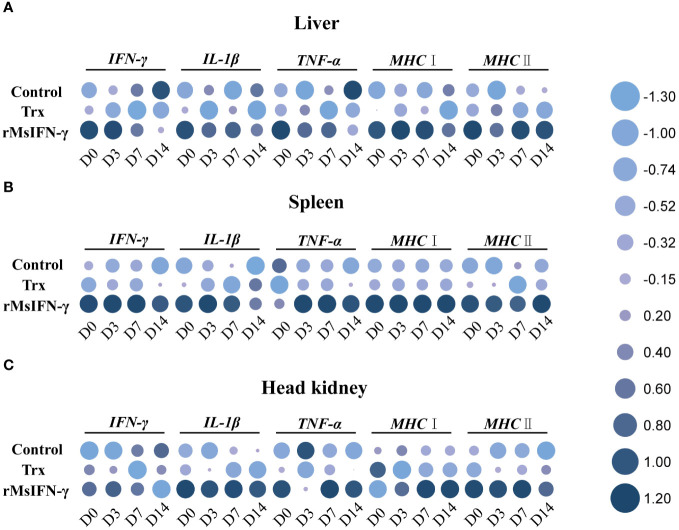
Relative expression of the immune-related genes in the liver **(A)**, spleen **(B)**, and head kidney **(C)** after infection on day 0 (D0), D3, D7, and D14. Data are shown as the means ± SEM (*n* = 3).

qRT-PCR was performed to identify the immune-related genes in the liver, spleen, and head kidney within 14 days following injection with bacteria. The results showed that, in the three examined tissues, the expression of the immune-related genes in the rMsIFN-γ group was higher and that the high expression appeared earlier (**Figure 6**). In the liver, the expression levels of IL-1β, MHC I, and MHC II in the control and Trx groups were all decreased within 14 days after bacterial stimulation, while the expression levels of IFN-γ and TNF-α were only significantly increased on D14 in the control group. In the rMsIFN-γ group, IFN-γ, IL-1β, and TNF-α peaked on D0 and D3 and then gradually decreased. MHC I and MHC II were highly expressed within 14 days after infection (**Figure 6A**). The spleen exhibited analogous patterns to the liver in terms of the results. Notably, in the control group, there was a reduction in the expression levels of the five immune-related genes. In contrast, in the rMsIFN-γ group, the duration of heightened expression for all five examined genes extended up to D14, indicating a prolonged and sustained immune response compared with the control and Trx groups (**Figure 6B**). Different from the observations in the liver and spleen, the head kidney displayed a distinct pattern of immune response. Specifically, within the rMsIFN-γ group, the expression of IFN-γ was consistently reduced across all examined time points, with a notable decrease observed on D14. In addition, the expression level of IL-1β in the head kidney was significantly elevated compared to that in the liver and the spleen. Moreover, the upregulation of MHC I expression manifested at a later stage, indicating a delayed immune response in the head kidney relative to the other tissues examined (**Figure 6C**).

### 3.6 Observation of histopathological changes

**Figure 7:**
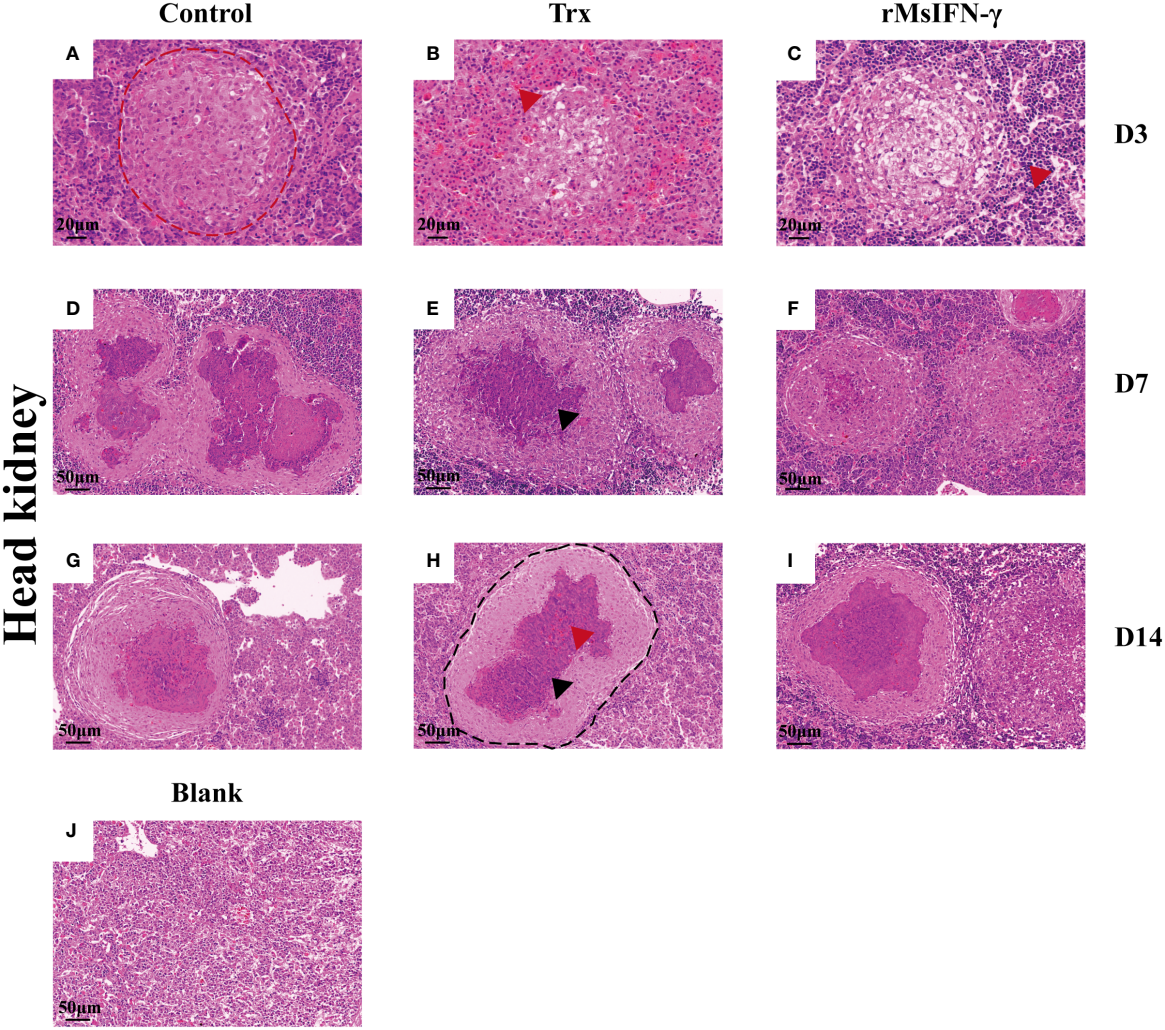
Histopathological changes in the head kidney. Representative images of the head and kidney on day 3 (D3), D7, and D14 after *Nocardia seriolae* infection in different treatment groups, stained with hematoxylin and eosin (HE). Red coils indicate early necrotic foci **(A)**, red arrows indicate bleeding **(B, I)** and inflammatory cells **(C)**; single granuloma spreads outward to form multiple granulomas **(D–F)**; the granuloma has multiple layers of thinly arranged connective tissue **(G)**; black coils indicate typical granulomatous structures **(H)**; black arrows indicate caseous necrosis within the granuloma **(E, I)**; healthy largemouth bass tissue was used as a blank control **(J)**.

**Figure 8:**
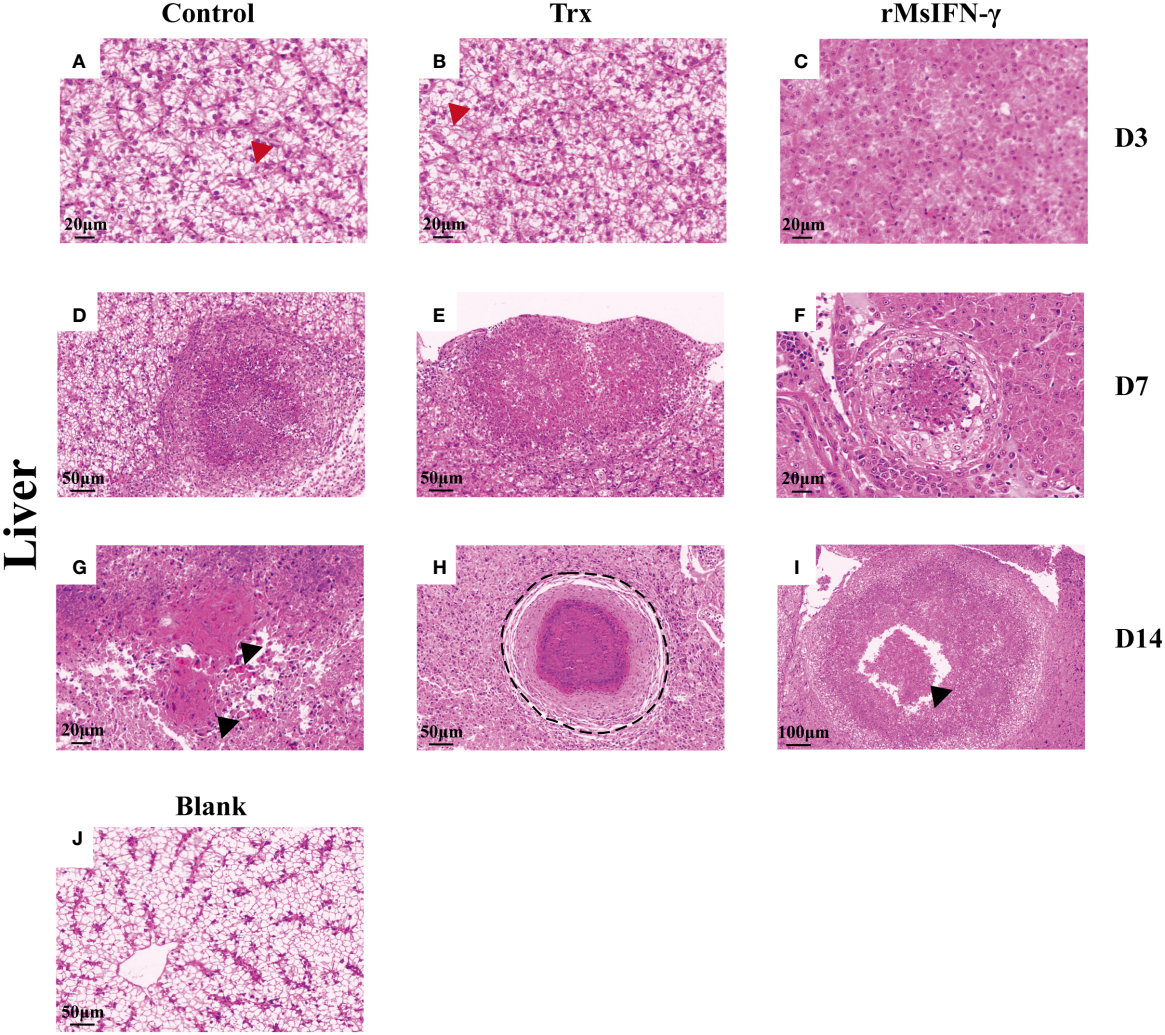
Histopathological changes in the liver. Representative images of the liver on day 3 (D3), D7, and D14 after Nocardia seriolae infection in different treatment groups, stained with hematoxylin and eosin (HE). Red arrows indicate cell swelling **(A, B)**; hepatocytes lacking lipids **(C)**; Granuloma of varying degrees of development **(D–F)**; black arrows indicate tissue fibrosis **(G)** and cellular debris **(I)**; black coils indicate typical granulomatous structures **(H)**; healthy largemouth bass tissue was used as a blank control **(J)**.

The liver and head kidney were chosen for histological investigation based on prior clinical observation. On D3, head kidney can be observed with obvious initial granuloma structure with necrotic foci filled with epithelioid cells (**Figure 7A**). Bleeding was also observed in head kidney (**Figures 7B, H**). Throughout granuloma development, inflammatory cells were continually recruited, and their aggregation in the D3 head kidney of the rMsIFN-γ group occurred much earlier than that in the control group (**Figure 7C**). As granuloma progresses, the necrotic foci underwent coagulation necrosis and formed a caseation (**Figures 7E, H**). Nevertheless, granulomas in the liver appeared later than in head kidney. Cellular swelling and vacuolar degeneration were seen in the liver on D3 (**Figures 8A, B**). Moreover, with decreased intracellular lipids in hepatocytes due to anorexia (**Figure 8C**), the liver cells differed significantly from those in the liver of healthy largemouth bass (**Figure 8J**). In the control group, fibrotic lesions (**Figure 8G**) and a distinct granulomatous trilaminar structure (**Figure 8H**) could already be seen. However, regardless of the length of infection, the head kidney and liver pathology evolution was slower in the rMsIFN-γ group than in the control group. Head kidneys and liver of normal healthy largemouth bass were used as blank controls (J in **Figures 7**, **8**). In conclusion, rMsIFN-γ can improve the survival rate after injection with *N. seriolae* by decreasing the bacterial load in tissues, boosting immune gene expression, and minimizing tissue damage.

## 4 Discussion

For *Nocardia*, numerous researchers have focused their efforts on histopathology studies and the isolation identification of pathogenic microorganisms. However, the mechanism of *N. seriolae* infection in the host remains less understood. It has been observed that the macrophages in tissues are primarily infected in the invasion pathway of *N. seriolae*. By inhibiting apoptosis, the survival of *N. seriolae* is achieved (24). In the present research, the cell wall composition of *N. seriolae* was found to be a rich and complex structure that contains peptidoglycans, lipids, and other substances (4, 25). Interestingly, this is comparable to the cell wall composition of *Mycobacterium tuberculosis* (Mtb) with both pathogenic bacteria containing cord factor, promoting the development of granulomas (26). Recent studies have also revealed that the lipid components of the cell walls of *N. seriolae* exhibit pathogenicity. This facilitates persistent infection of the bacteria (27). Thus, for further study on the pathogenic mechanism and therapy of *N. seriolae*, it is therefore possible to refer to the mechanism of action of Mtb. As is widely known, IFN-γ plays an essential role in the formation of granulomas and the control of multiple pathogens. It mainly targets intracellular bacteria, including Mtb (28) and *Listeria monocytogenes* (29). IFN-γ was able to promote the differentiation of Th1 cells, which are key to cellular immunity to bacterial infections within cells (30). Based on this information, we investigated the action of MsIFN-γ in the control of *N. seriolae* infection in largemouth bass.

In the present study, the MsIFN-γ of largemouth bass was identified by genome-wide alignment. The distinctive sequence ([I/V]-Q-X-[K/Q]-A-X2-E-[L/F]-X2-[I/V]) and relatively conserved NLS sequences were discovered at the C-terminus of the MsIFN-γ sequence, consistent with the IFN-γ of other Perciformes species. These features are not shared by IFN-γrel, which does not have a conserved NLS sequence. As proven in both rainbow trout (*Oncorhynchus mykiss*) (31) and humans (32), the deletion of NLS sequences causes loss of the biological activity of IFN-γ. This might indicate that MsIFN-γ could participate in the immune regulation of the organism.

IFN-γ was found in many teleosts, but the level of expression varied slightly. In our studies, MsIFN-γ was mostly expressed in the blood, followed by the gill and the heart. The basic expression level of MsIFN-γ in tissues showed the lowest in the spleen. Strong evidence exists that the blood is important in the transport of nutrients, hormones, proteins, and cells throughout an organism (33). The heart is the principal organ promoting blood circulation in the whole body. The high expression of MsIFN-γ in tissues could contribute to the transmission of signals to immune cells throughout the body and activate the immune system. When an infection occurs, immunological cells are rapidly triggered to release a variety of immune substances to combat them. In addition, the gills serve as the primary organ in contact with the external environment. Therefore, it was thought to be the entrance of pathogenic bacteria into the fish body in the aquatic environment (34). The large number of immune cells contained in the gills was also thought to be able to react rapidly to MsIFN-γ responses (35). Currently, the expression levels of type II IFNs have been assessed in a variety of fish species, including the Atlantic cod (*Gadus morhua*) (36), Atlantic halibut (*Hippoglossus hippoglossus* L.) (37), carp (*Cyprinus carpio* L.) (38), and goldfish (39). Expression of the IFN-γ of the large yellow croaker was discovered to be greater in the blood than in other tissues. On the other hand, type II IFNs are highly expressed mainly in immunological organs, where they play important roles in the immune system.

For monocytes/macrophages, IFN-γ is the most important activated molecule (40). It induces monocytes/macrophages to M1 polarization and secretes a variety of pro-inflammatory cytokines (41). When the monocytes/macrophages of largemouth bass were treated with rMsIFN-γ, the expression levels of the pro-inflammatory cytokines (e.g., IL-1β, TNF-α, and IL-6) were enhanced. This, in addition to IFN-γ, boosted the macrophage phagocytosis and microbicidal activity. An example is the production of reactive nitrogen and oxygen, including the formation of superoxide radicals, nitric oxide, and hydrogen peroxide (42). The zebrafish (*Danio rerio*) has been used to illustrate the role of TNF-α in the removal of Mtb (43). The increased expression of IL-6 led to an increase in the expression of Hep-1, which can effectively regulate the iron metabolism in the body. Similarly, the expression levels of MHC I and MHC II were also elevated. Antigen-presenting cells (APCs) could deliver antigen-bound MHC I and MHC II to CD8^+^ and CD4^+^ T cells, producing humoral immunity and cellular immunity. Our findings are in line with those of previous studies on grass carp and large yellow croaker (44). The above evidence demonstrates that rMsIFN-γ has a clear activation effect on the monocytes/macrophages of largemouth bass *in vitro.* Thus, monocytes/macrophages constitute a powerful defense in the face *N. seriolae* invasion.

The survival rate was determined by intraperitoneal injection of rMsIFN-γ in a model of *N. seriolae* infection in largemouth bass. The survival rates of the rMsIFN-γ group were higher by 42.10% and 27.63% than those in the control group. Regarding the clinical symptoms and the bacterial load in tissues, the rMsIFN-γ-treated group was clearly ameliorated compared with the control group. The spread of *N. seriolae* is related to blood circulation, with the most pronounced symptoms in the liver, spleen, and head kidney (45). The spleen and head kidney, as the main hematological and immunological organs of teleost fish, are rich in lymphocytes and macrophages (46). The liver plays a vital role in fish digestion and metabolism. Previously, treatment with recombinant IFN-γ in bastard halibut (*Paralichthys olivaceus*) and ginbuna crucian carp ( *Carassius auratus* langsdorfii) was effective against intracellular bacterial invasion (*Edwardsiella tarda* and *N. seriola*, respectively) (47, 48). Moreover, IFN-γ knockout mice were more susceptible to bacterial diseases (49, 50).

In order to further explore the effect of rMsIFN-γ *in vivo*, the cytokine expression levels and tissue damage in the liver, spleen, and head kidney were examined. rMsIFN-γ treatment increased the cytokine expression level compared with the control group. Obvious changes were observed in the inflammatory cytokines (IL-1β and TNF-α) after treatment. In addition, after booster injection of rMsIFN-γ, we found that the expression levels of the pro-inflammatory cytokines (IL-1β and TNF-α) in immune tissue were maintained at high levels for a long time. These signals prove the monocyte/macrophage M1 polarization toward the host, accelerating bacterial clearance. This evidence is also in line with the *in vitro* experiments. The primary target cells were the monocytes/macrophages after *N. seriolae* infection in fish. Inflammatory activation of monocytes/macrophages was required for effective phagocytosis and clearance of invading *N. seriolae*. However, this process could produce antigenic peptides to bind MHC II, which can activate CD4^+^ T cells to undergo Th0 cell differentiation into Th1 (51). Th1 cells are capable of releasing cytokines (e.g., IFN-γ and TNF-α), which further affects the local inflammatory response and activated macrophages. It plays a significant role in the resistance of intracellular bacteria such as *Mycobacteria* and *Brucella* (52). Similarly, CD8^+^ T cells differentiate through the antigen presentation of MHC I and IL-2 released by Th1 cells. Subsequently, cytotoxic T lymphocytes (CTLs) are produced, which participate in the killing of intracellular bacteria. To eliminate *N. seriolae* infection, cellular immunity should be able to dominate the overall defensive mechanism, with humoral immunity acting as an additional defense. These processes together facilitate the goal of eliminating *N. seriolae* in monocytes/macrophages.

Interestingly, the effectiveness of rMsIFN-γ in reducing *N. seriolae* infection is supported by histological findings. According to our research, the condition of inflammatory granulomas varied during the *N. seriolae* infection period. Granulomatous structures have been previously reported (53). Early granulomas consist of epithelial cells differentiated from monocytes to form a focus of necrosis, with the diameter of granuloma gradually increasing over time. There are more inflammatory cells around the granuloma, including monocytes, lymphocytes, and macrophages, among others. In the middle stage, inflammatory cells and *Nocardia* invade the granuloma, causing the cells to be fragmented and coagulated necrosis to ensue. Granulomas showed a typical three-layer structure, from the inner to the outer structure in the following order: necrotic cellular debris, sparse connective tissue, and an epithelial cell layer. The presence of bacteria could also be seen in the granuloma by HE staining. During this stage, the diameter of the granuloma steadily expanded, as did the amount of caseous necrosis, which was accompanied by the outward movement of the granuloma. The epithelial cell layer wraps around the freshly created small granuloma, forming two or more connected granulomas. As the disease progresses, calcium salts are deposited in the necrotic foci and bacteria surrounded by peripheral fibroblasts cannot be clearly observed. Finally, part of the granuloma is formed by fibroblasts into connective tissue. The granuloma caused by *N. seriolae* and Mtb are not much different, but multinucleated giant cells can be seen only in Mtb-infected tissues (54). In the rMsIFN-γ treatment group, the rate of granuloma formation was slowed down and the number of inflammatory cells was increased. This supports that the rMsIFN-γ of largemouth bass could deal with *N. seriolae* infection more effectively.

In conclusion, the pathogenesis and treatment methods for *N. seriolae* infection are inadequate, and further detailed studies are needed. In this study, we used IFN-γ as a broad-spectrum and highly efficient cytokine to achieve effective treatment of *Nocardiosis*. It activates the immune system by activating the body’s monocytes/macrophages and releasing inflammatory factors. This research demonstrates the great potential of cytokines in the treatment of bacterial diseases, providing a new direction for the green development of aquaculture.

## 5 Conclusion

The MsIFN-γ was cloned in this work, demonstrating that its sequence was mostly preserved and related to other Perciformes fish. It has been illustrated that rMsIFN-γ is physiologically active and activates monocytes/macrophages for immunomodulatory effects at the cellular and individual levels. In order to better understand the differences in clinical symptoms and the pathological changes caused by *N. seriolae* infection in largemouth bass, rMsIFN-γ was utilized. The model also revealed how the rMsIFN-γ boosted the body’s immune response and lessened the rate of tissue destruction.

## Data availability statement

The original contributions presented in the study are included in the article/**Supplementary Material**. Further inquiries can be directed to the corresponding author.

## Ethics statement

All procedures of animal experiments were reviewed and approved by the Ethical Committee on Animal Research at Huazhong Agricultural University (ID Number: HZAUFI-2022-0015). All the efforts were made to minimize animal suffering. The study was conducted in accordance with the local legislation and institutional requirements.

## Author contributions

RY: Conceptualization, Data curation, Investigation, Methodology, Project administration, Software, Validation, Writing – original draft, Writing – review & editing. WZ: Formal analysis, Methodology, Project administration, Supervision, Validation, Writing – original draft. PY: Investigation, Methodology, Software, Supervision, Validation, Writing – original draft. JZ: Formal analysis, Investigation, Software, Supervision, Validation, Visualization, Writing – original draft. JS: Writing – review & editing. GY: Conceptualization, Formal analysis, Funding acquisition, Investigation, Project administration, Resources, Software, Supervision, Visualization, Writing – original draft, Writing – review & editing.
