# Supplementary material for: IFN-γ enhances protective efficacy against Nocardia seriolae infection in largemouth bass (Micropterus salmoides)
Source: Front Immunol. 2024 Mar 13;15:1361231. doi: 10.3389/fimmu.2024.1361231 (PMC10965728; doi:10.3389/fimmu.2024.1361231)
Supplement: Supplementary file 1 [file DataSheet_1.docx]

# Supplementary Figures


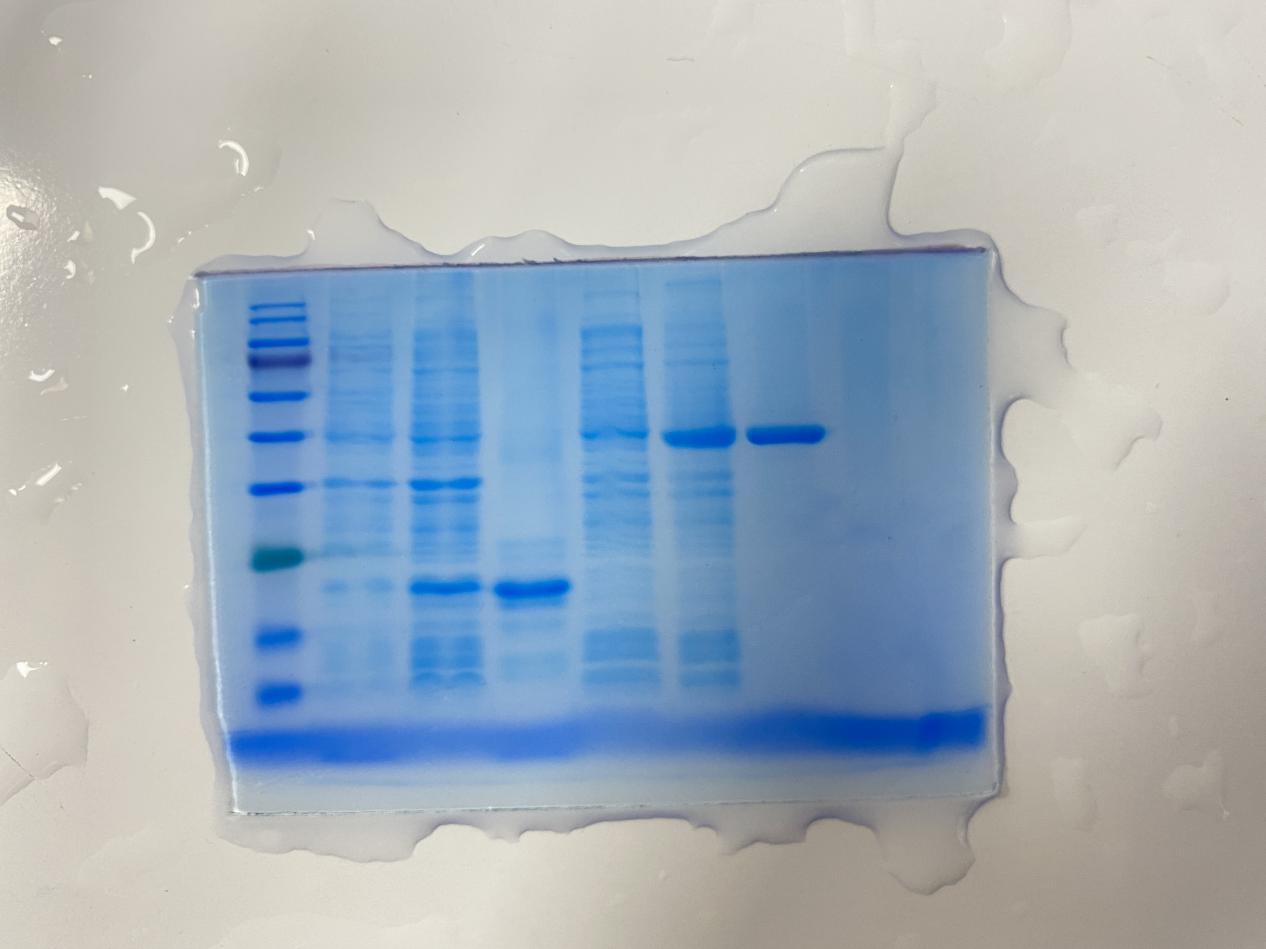


**M 1 2 3 4 5 6**

This SDS-PAGE assay result is the original image of Fig 3B. Lane M: protein molecular weight marker (10-180kDa); Lane 1:. Uninduced pET-32a protein; Lane 2. Induced pET-32a protein; Lane 3. Purified Trx protein; Lane 4. Uninduced protein of pET-32a-MsIFN-γ; Lane 5. Induced pET-32a-MsIFN-γ protein; Lane 6: Purified MsIFN-γ protein.

***
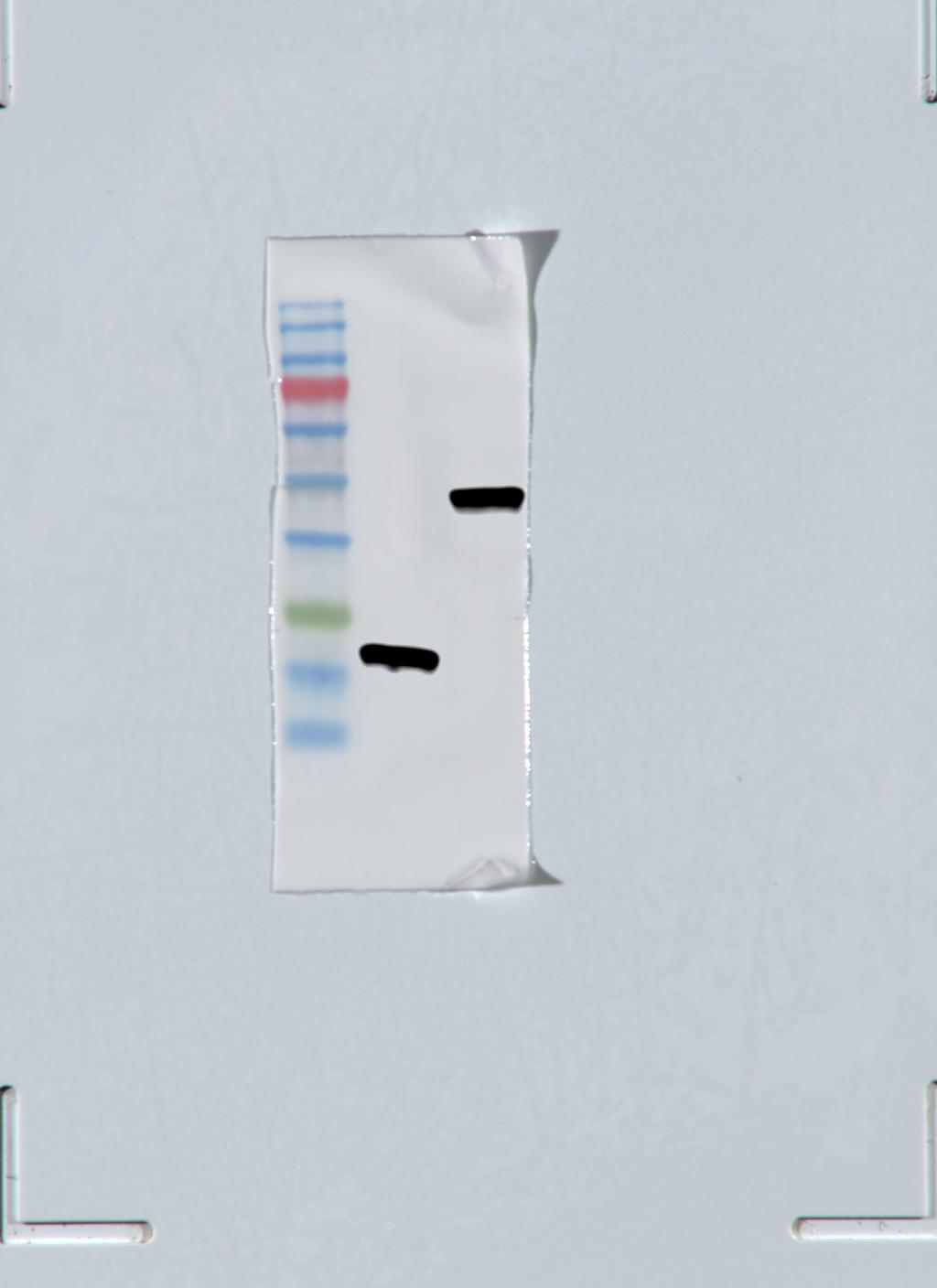
***

**M 1 2**

This WB assay result is the original image of Fig 1B. M: Standard protein molecular weight Maker; 1. Purified Trx protein; 2. Purified rMsIFN-γ protein.
