# Supplementary material for: IFN-γ enhances protective efficacy against Nocardia seriolae infection in largemouth bass (Micropterus salmoides)
Source: Front Immunol. 2024 Mar 13;15:1361231. doi: 10.3389/fimmu.2024.1361231 (PMC10965728; doi:10.3389/fimmu.2024.1361231)
Supplement: Supplementary file 2 [file Table_1.docx]

# Supplementary Tables

Table S1 primers sequences used in the current study.

| **Gene** | **Application** | **Primer name** | **Oligonucleotide sequence (5′-3′)** | **GeneBank** |
| --- | --- | --- | --- | --- |
| hep-1 | qRT-PCR | Forward | CAGGGAGCAATGACACTCCA | EU502749.1 |
|  |  | Reverse | CTCCGTGGGGAAACACATCC |  |
| IFN-γ | [Expression](javascript:;) | Forward | CCGCTCGAG(*Xho*Ⅰ)GACTCTCAGATGAGTTTTGGC | XM_038707474.1 |
|  |  | Reverse | CGGGATCC(*BamH*Ⅰ)TTTCAAATCCCTCTGAAGATG |  |
|  | qRT-PCR | Forward | AAAACGCCACCCATAAACACC |  |
|  |  | Reverse | CAAATCCCTCTGAAGATGAACAAA |  |
| IL-1β | qRT-PCR | Forward | CGTGACTGACAGCAAAAAAGAGG | (Byadgi et al., 2016) |
|  |  | Reverse | GATGCCCAGAGCCACAGTTC |  |
| IL-6 | qRT-PCR | Forward | ATTTCCCGAGAACTGAGGACTAAC | XM_038732985.1 |
|  |  | Reverse | TTTCACTGATGTACCAGCCACCT |  |
| MHC Ⅰ | qRT-PCR | Forward | GTGGTTCAACGTCAACATCG | (Hoang et al., 2020) |
|  |  | Reverse | ACCCAGACTTGTTCGGTGTC |  |
| MHC Ⅱ | qRT-PCR | Forward | GAGGACCTTGCTGTCATTGG | (Hoanget al., 2020) |
|  |  | Reverse | GCGTACCAAACCTCTTCACC |  |
| *N. seriolae* 16s rRNA | qRT-PCR | Forward | TGCTACAATGGCCGGTACAGAG | (Wang et al., 2012) |
|  |  | Reverse | TTCACGAGGTCGAGTTGCAGAC |  |
| TNF-α | qRT-PCR | Forward | CTTCGTTCTACAGCCAGGCATCG | (Byadgiet al., 2016) |
|  |  | Reverse | TTTGGCACACCGACCTCACC |  |
| β-actin | qRT-PCR | Forward | CCACCACAGCCGAGAGGGAA | (Byadgiet al., 2016) |
|  |  | Reverse | TCATGGTGGATGGGGCCAGG |  |

In the table, the nucleotides lowercase letters indicate the positions of the restriction enzyme sites. “Expression” represents that the primer is used for vector construction; “qRT-PCR” represents that the primer is used to analyze gene mRNA expression level. F, forward; R, reverse.
